# Comparison of the effect of rapeseed oil or amaranth seed oil supplementation on weight loss, body composition, and changes in the metabolic profile of obese patients following 3-week body mass reduction program: a randomized clinical trial

**DOI:** 10.1186/s12944-020-01330-7

**Published:** 2020-06-20

**Authors:** Małgorzata Moszak, Agnieszka Zawada, Aldona Juchacz, Marian Grzymisławski, Paweł Bogdański

**Affiliations:** 1 Department of Obesity and Metabolic Disorders Treatment and Clinical Dietetics, Karol Marcinkowski University of Medical Sciences, ul. Szamarzewskiego 82/84, 60-569 Poznań, Polska Poland; 2 Department of Gastroenterology, Dietetics and Internal Medicine, Karol Marcinkowski University of Medical Sciences, Poznan, Poland; 3 Centre of Pulmonology and Thoracic Surgery, Poznan, Poland

**Keywords:** Amaranth seed oil, Body composition, Metabolic profile, Rapeseed oil, Weight loss

## Abstract

**Background:**

Amaranth seed oil (ASO) and rapeseed oil (RSO) are functional foods that display antioxidant and hepatoprotective properties. These oils are also known to lower glucose and cholesterol levels. The current study compared the effects exerted by RSO and ASO on weight loss and metabolic parameters during a 3-week body mass reduction program.

**Methods:**

Eighty-one obese subjects (BMI > 30 kg/m^2^), aged 25–70 years, were enrolled in a 3-week body mass reduction program based on a calorie-restricted diet and physical activity. Participants were randomly categorized into an AO group (administered 20 mL/d of ASO), a RO group (administered 20 mL/d of RSO), and a C group (control; untreated). Anthropometric and metabolic parameters were measured at baseline and endpoint.

**Results:**

Significant decreases in weight, body mass index (BMI), waist circumference (WC), hip circumference (HC), fat mass (FM), lean body mass (LBM), visceral fat mass (VFM), and total body water (TBW%) were observed in all groups (*P* <  0.05). No significant improvements were observed in the clinical parameters of group C. Fasting insulin (Δ − 5.9, and Δ − 5.7) and homeostatic model assessment of insulin resistance (HOMA-IR) (Δ − 1.1 and Δ − 0.5) were decreased in both RO and AO groups, respectively. Fasting glucose (Δ -8.5; *P* = 0.034), total cholesterol (Δ -14.6; *P* = 0.032), non-HDL cholesterol (Δ 15.9; *P* = 0.010), TG/HDL ratio (Δ -0.6; *P* = 0.032), LDL cholesterol (Δ -12.3; *P* = 0.042), and triglycerides (Δ -6.5; *P* = 0.000) were significantly improved in the AO group, compared to the RO group.

**Conclusions:**

The 3-week body mass reduction intervention caused a significant reduction in the weight, BMI, WC, HC, FM, and VFM of all groups. Except for HOMA-IR, there were no statistical differences between the clinical parameters of all groups. However, a trend toward improved insulin levels and HDL% was noticeable in AO and RO. Therapies involving edible oils with high nutritional value, such as RSO and ASO, show potential for improving metabolic measurements during body mass reduction programs. Thus, obese patients undertaking weight reduction programs may benefit from RSO and ASO supplementation.

**Trial registration:**

retrospectively registered, DRKS00017708.

## Background

Obesity, which is a significant public health issue, has reached pandemic levels in the developed world [1]. According to 2016 World Health Organization (WHO) statistics, 39% of the population was overweight and 650 million (13%) were obese [2]. Lifestyle changes which focus on proper nutrition and physical activity constitute the primary approach for treating obesity [3]. Functional food consumption has been suggested as another useful method for reducing the prevalence of obesity. Functional foods are defined as foods fortified with usually scarce nutrients or foods without harmful ingredients (e.g. allergens), which provide health benefits. For example, Cicero et al. [4] described lipid- and blood pressure-lowering properties of bioactives such as berberine, plant sterols, green tea extract, soy, curcumin, cocoa, pycnogenol, lycopene, olive oil, soluble fibers, garlic, resveratrol, beetroot, and mineral salts. Although functional food products deliver additional or enhanced benefits over and above their basic nutritional value, these foods should not be considered as alternatives to a balanced diet [5, 6].

Amaranth seed oil (ASO) and rapeseed oil (RSO) are functional food products that are becoming increasingly popular. The health-promoting properties of both oils are mainly due to a high content of monounsaturated fatty acids (MUFAs) and polyunsaturated fatty acids (PUFAs). In addition, properties of both oils are determined by a unique composition of bioactive substances as follows: squalene, sterols, tocopherols, carotenoids, phospholipids, etc., for ASO; and tocopherols, carotenoids, flavonoids, phytosterols, and phenolic links (sinapine) for RSO [7, 8]. However, ASO and RSO differ in popularity, availability and price. RSO is one of the most popular and commonly used oils in Poland, while ASO is considered to be “exotic” and “healthier” than RSO [9].

Previous studies have indicated that diets supplemented with ASO improved antioxidant properties, and exhibited anti-inflammatory, hypotensive, and hepatoprotective effects [10]. Furthermore, Kim et al. [11] investigated the effect of amaranth seed and amaranth oil supplementation on the blood glucose profiles of streptozotocin-induced diabetic rats. The hypoglycemic activity of ASO has not yet been established via clinical trials. RSO, which also possesses similar antioxidant, hypolipemic, anti-inflammatory and anti-atherogenic properties, is cheaper and more ubiquitous than ASO [12, 13]. Previous studies have shown that modulation of a fatty acid profile by converting fats commonly present in diet, such as RSO, has a positive effect on carbohydrate metabolism and reduces the risk of developing breast cancer [7].

However, studies comparing the effectiveness of ASO and RSO on weight reduction and normalization of metabolic parameters associated with obesity are lacking. Fatty acid compositions of ASO and RSO are different. RSO contains higher levels of MUFAs and PUFAs than ASO and displays a more beneficial PUFA/saturated fatty acid (SFA) ratio and unsaturated fatty acid (USFA)/SFA ratio than those of ASO. However, ASO is rich in squalene [14, 15]. The fatty acid composition of both oils is shown in Table 1.

**Table 1:** Fatty acid composition of ASO and RSO

Component	Amaranth seed oil	Rapeseed oil
16:0 (palmitic acid) SFA	18.5–23.4%	2.9%
16:1 (palmitoleic acid)	0.09–0.4%	0.2%
18:0 (stearic acid) SFA	3.4–4.5%	4.5%
18:1 n-9 (oleic acid) MUFA	22.6–26.0%	59%
18:2 n-6 (linoleic acid) PUFA omega 6	38.2–49.9%	21%
18:3 n-3 (α-linolenic) PUFA omega 3	0.92–1.2%	11.2%
20:1 n-9 (eicosenoic acid)	0.1	1.4%
22:0 (behenic acid)	0.1–0.4%	2%
22:1 (euric acid)		0.1%
24:0 (lignoceric acid)	0.1–0.4%	1.5%
Squalene	58.8–77.7 mg/g	< 0.05 mg/g
Phytosterols	1931–2762 mg/100 g	558–1407 mg/100 g
Tocopherols	656.8–2588 mg/kg	620–950 mg/kg

The objective of this study was to compare the effect of ASO and RSO supplementation on anthropometric parameters (body mass, body mass index (BMI), waist circumference (WC), hip circumference (HC), waist/hip ratio (WHR), and body composition) and selected biochemical parameters (fasting serum glucose and insulin levels, homeostatic model assessment of IR (HOMA-IR), total cholesterol (TC), high-density lipoprotein (HDL), low-density lipoprotein (LDL), triglycerides (TG), non-HDL levels and TG/HDL ratios in obese adults following a strictly controlled weight loss program lasting 3-weeks.

## Methods

### Research design

The study was designed as a randomized, double-blind, controlled trial with 3 parallel groups, and performed at the Department of Gastroenterology, Internal Diseases and Dietetics, from July 2014 to March 2016. The study protocol was approved by the Research Ethics Committee of the Poznan University of Medical Sciences, Poland (approval 333/14), and the study was performed in accordance with the Helsinki Declaration. All patients provided signed informed consent prior to participation in the study. The study was retrospectively registered in the Deutsches Register Klinischer Studien under the number DRKS00017708.

### Participants

**Fig. 1:**
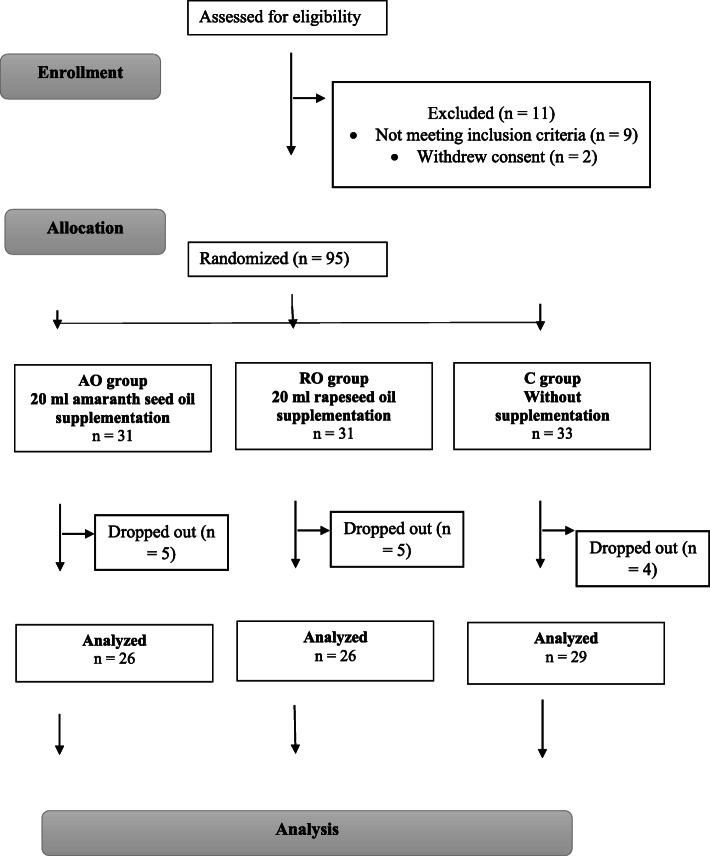
Study flowchart

One hundred and six obese Polish adults were enrolled. Inclusion criteria for the study were BMI ≥ 30 kg/m^2^, stable body weight (< 3 kg self-reported change during the previous 3 months) and being 18–70 years old. The exclusion criteria were as follows: (i) secondary form of obesity; (ii) chronic diseases related to metabolism (e.g., chronic liver, kidney, pancreas diseases, inborn metabolic diseases, autoimmune diseases, inflammatory bowel diseases, coeliac disease); (iii) diabetes mellitus type 1 or uncontrolled diabetes mellitus type 2; (iv) uncontrolled disorders of lipid metabolism; (v) vegetarian or any another alternative dietary habit; (vi) a history of using any dietary supplements within the 3 months prior to the study; (vii) diagnosed with hyperlipidemia or hypertension, or diabetes mellitus type 2, or requiring the introduction and/or change of pharmacological treatment for these diseases during the 2 years before the trial; (viii) eating disorders; and (ix) nicotine, alcohol or drug abuse. Prior to randomization, 9 subjects were excluded according to exclusion criteria, while 2 withdrew consent. The remaining 95 patients were randomized and subjected to intervention. During the course of the 3-week period, 14 patients failed to complete the study as follows: 4 from C, 5 from AO, and 5 from RO. Finally, data pertaining to 81 participants were analyzed. Among those analyzed, 49 had hypertension, 27 had type 2 diabetes and 35 had hyperlipidemia. However, these subjects maintained a constant pattern of pharmacotherapy in accordance with exclusion criteria. The flowchart of the study is shown (Fig. 1).

### Interventions

Each eligible and consenting participant was assigned a unique code as an identifier. The study population was randomly assigned to 3 treatment groups as follows: 20 mL ASO (AO group; 16 women, 10 men); 20 mL RSO (RO group, 16 women, 10 men); and untreated (C group (control); 19 women, 10 men). Participants and investigators were blinded to randomization and group allocation. Patients from all groups underwent a 3-week body mass reduction program conducted under controlled conditions. The duration of the study was determined to be 3-weeks due to the need to hospitalize participants for the entire intervention period. Thus, the body mass reduction program was implemented during a 21-day hospital stay. The study participants did not have an opportunity to meet people other than medical staff or obtain food except for the meals provided by a specialized food caterer.

During the 3-week body mass reduction program, each subject received daily physical training, in the form of aerobics, from a physical therapist as well as a hypocaloric diet (70–75% of the total daily energy expenditure (TDEE)). The TDEE was individually tailored for each participant using the Harris-Benedict formula and the physical activity level (PAL) index. In order to determine the TDEE and assess the real energy value of the diet, before the program, subjects were required to complete a 24-h record for 3 days. All subjects received the same type of diet prepared by a dietetic food caterer based on a planned menu. Each patient received a diet with an identical composition of macronutrients (20% protein, 25–30% fat, and 50–55% carbohydrates) derived from the same products. The diets were supplemented with 20 mL ASO (AO group) or 20 mL RSO (RO group) per day instead of the 20 g of fat (butter) normally included in the diet. One meal (breakfast) was supplemented with one dose of oil. ASO “Ol’Amar” produced by “Szarłat” (Lomza, Poland; cold-pressed amaranth oil containing squalene (5.0–6.5 g/100 mg) and vitamin E (5–10 mg/100 g) and RSO produced by “Ol’Vita (Marcinowice, Poland; cold-pressed rapeseed oil containing omega 9 (60%), vitamin E (66.4 mg / 100 g) at a n-6:n-3 ratio of 2.5:1) were used. Each dose of the oil supplement and the meal was delivered to the participant by a nurse. Compliance with the study protocol was assessed daily by a nurse and weekly by a dietician.

Physical training included active and passive breathing exercises for 30 min a day, cardiovascular aerobic exercises twice daily for 60 min and resistance training for 30 min daily. The aerobic training session consisted of a 5 min warm-up session (stretching exercises) at low intensity, followed by 45 min of training (Nordic walking or cycling) at an intensity between 50 and 70% of the maximum heart rate, and 5 min of closing stretching and breathing exercises of low intensity. Resistance training consisted of a 5 min warm-up session (stretching exercises) of low intensity, followed by 20 min of exercises with a neck barbell. Kettlebells and gymnastic ball and a 5 min warm-up session (stretching exercises) at low intensity.

### Anthropometric assessment

Anthropometric and metabolic parameters were assessed at baseline and endpoint for each patient. WC (cm) was measured at the midpoint between the lower margin of the least palpable rib and top of the iliac crest, while HC was measured around the widest portion of the buttocks with the tape held parallel to the floor. Both measurements were performed using stretch-resistant medical tape (Seco) and repeated twice to obtain a certain result. Height was measured to the nearest centimeter using a stadiometer. Weight and body composition were assessed using bioelectrical impedance, Tanita MC 980 MA (Tanita, Tokyo, Japan). Body composition analysis was performed in accordance with the recommendations of the European Society for Clinical Nutrition and Metabolism (ESPEN) [16]. BMI (kg/m^2^) was defined as the individual’s body mass divided by the square of their height. WHR was estimated as the ratio of WC to the HC.

### Clinical assessment

Biochemical and anthropometric measurements were made at the baseline and endpoint. Fasting venous blood samples (10 mL) were collected at the beginning and the end of the program. Venous blood samples (10 mL) were collected by qualified nurses. Collection was performed between 8:00 and 10:00 am under conditions of fasting after overnight resting tubes containing biological material were coded according to the system used. In order to obtain serum, blood was centrifuged at 1500×*g* for 10 min and preserved in 2 mL Eppendorf tubes. Serum glucose, TC, HDL, and TG levels were analyzed using a fully automated Modular P-800 Roche (Diamond Diagnostics, Budapest, Hungary). LDL was indirectly measured using the Friedewald equation as follows: LDL (mg/dL) = TC – HDL – (TG/5) [17]. Fasting insulin levels were determined via a microparticle enzyme immunoassay (Abbot, Abbot Park, IL, USA). The sensitivity of the assay, as reported by the manufacturer, was represented by a mean minimum detectable value of 1.0 μU/mL. HOMA-IR was calculated using the following formula: fasting insulin (μU/L) × fasting glucose (nmol/L)/22.5 [18].

### Statistical analysis

Statistical analyses were performed using Statistica 10.0 (StatSoft) and the MetaboAnalyst 3.0. server (www.metaboanalyst.ca). Normality of data was checked using the Shapiro-Wilks test. Normally distributed data are expressed as mean ± SD, while skewed data are represented by the median (interquartile range). Differences among the 3 groups were determined using the Kruskal–Wallis test followed by an appropriate post-hoc test (multiple comparison test) or one-way ANOVA followed by Tukey’s post-hoc test for normally distributed data. In order to test the differences between the baseline and endpoint in each group, the Wilcoxon test or paired *t*-test (for normally distributed data) was conducted [19]. Statistical significance was set at *P* <  0.05 [20].

## Results

Eighty-one obese patients (mean BMI: 39.6 ± 7.4 kg/m^2^) were enrolled in the study. Baseline anthropometric and biochemical characteristics of AO, RO and C groups are shown (Tables 2 and 3). The differences between baseline metabolic and anthropometric variables of the 3 groups were not significant.

**Table 2:** Baseline anthropometric characteristics of RO, AO, and C groups

Variable	RO group (***n*** = 26)	AO group (***n*** = 26)	C (***n*** = 29)	***P***-value
Weight, kg	120.5 (102.9–135)	118.5 (108.0–132.4)	115.0 (101.4–133.3)	0.823^b^
BMI, kg/m^2^	39.8 (36.9–44.0)	40.6 (36.7–44.3)	38.4 (36.1–41.7)	0.490^b^
WC, cm	126.3 ± 13.3	124.3 ± 17.2	122.3 ± 20.6	0.600^a^
HC, cm	128.0 ± 13.3	130.6 ± 12.9	119.9 ± 20.6	0.113^a^
WHR	0.99 ± 0.09	0.95 ± 0.1	1.02 ± 0.06	0.112^b^
FM, %	41.0 ± 5.4	42.1 ± 6.1	40.9 ± 6.7	0.641^a^
FM, kg	46.9 (41.9–56.2)	49.7 (41.0–59.6)	46.7 (40.4–54.1)	0.594^b^
LBM, kg	66.9 (58.6–75.6)	68.7 (56.5–77.2)	70.1 (58.5–80.4)	0.810^b^
VFM, n	15.0 (12.0–21.0)	14.0 (13.0–21.0)	16.0 (12.0–20.0)	0.971^b^
TBW, %	41.6 (40.3–44.5)	41.4 (38.7–44.7)	41.8 (39.0–44.9)	0.792^b^
TBW, kg	49.1 (43.7–58.9)	50.6 (42.6–58.1)	48.6 (41.6–57.0)	0.783^b^
ECW, kg	22.7 ± 2.9	23.1 ± 4.2	22.1 ± 3.5	0.700^b^
ICW, kg	25.6 (23.0–32.9)	27.9 (22.5–33.5)	26.0 (21.4–31.8)	0.800^b^

Normally distributed data are represented by the mean ± SD and skewed data by the median (interquartile range). *P*-value < 0.05 is considered significant

^a^one-way ANOVA, ^b^Kruskal-Wallis test,

*Abbreviations*: *BMI* body mass index, *ECW* extracellular water, *FM* fat mass, *HC* hip circumference, *ICW* intracellular water, *LBM* lean body mass, *TBW* total body water, *VFM* visceral fat mass, *WC* waist circumference, *WHR* waist-to-hip ratio

**Table 3:** Baseline clinical characteristic of RO, AO, and C groups

Variable	RO group (***n*** = 26)	AO group (***n*** = 26)	C (***n*** = 29)	***P***-value
Age	50.7 ± 13.5	46.6 ± 10.4	49.9 ± 13.4	0.273^a^
Fasting plasma glucose, mg/dL	105.0 (98.0–119.0)	110.5 (95.0–131.0)	104.0 (98.0–114.0)	0.852^b^
Fasting insulin, μU/L	18.5 (11.3–29.3)	22.6 (13.2–27.5)	15.5 (13.0–22.2)	0.591^b^
HOMA-IR	4.8 (2.9–7.5)	6.1 (3.2–8.9)	4.0 (3.3–5.3)	0.410^b^
TC, mg/dL	196.6 ± 39.5	193.8 ± 37.0	192.5 ± 47.8	0.944^a^
HDL, mg/dL	48.0 (41.0–66.0)	42.5 (37.0–48.0)	49.0 (42.0–57.0)	0.060^b^
HDL, %	29.2 (19.0–33)	21.1 (18.0–29.6)	26.0 (22.0–32.0)	0.191^b^
non-HDL, mg/dL	143.5 ± 39.8	149.6 ± 39.7	149.1 ± 43.9	0.773^a^
TG/HDL ratio	2.3 (1.4–4.0)	3.7 (2.4–5.4)	2.9 (2.0–5.6)	0.090^b^
LDL, mg/dL	121.5 ± 33.9	117.6 ± 35.1	108.0 ± 36.6	0.484^a^
TG, mg/dL	97.0 (79.0–177.0)	143.0 (103.0–204.0)	142.0 (104.0–196.0)	0.082^b^

Normally distributed data are represented by mean ± SD and skewed data by the median (interquartile range). *P* < 0.05 is considered significant

^a^one way ANOVA, ^b^Kruskal-Wallis test,

*Abbreviations*: *HDL* high-density lipoprotein cholesterol, *HOMA-IR* homeostatic model assessment of insulin resistance, *LDL* low-density lipoprotein cholesterol, *TC* total cholesterol, *TG* triglycerides, SI conversion factors: to convert TC, HDL, and LDL to mmol/L, multiply by 0.02586; TG to mmol/L, by 0.0114; glucose to mmol/L, by 0.05551

Statistically significant (*P* < 0.05) decreases in body weight, BMI, WC, HC, FM, LBM, VFM, and TBW% were observed in all study groups following the 3-week body mass reduction program. There were significant differences between the changes caused by intervention in WC (*P* = 0.022), HC (*P* = 0.007) and VFM (*P* = 0.020) of the RO, AO, and C groups. (Table 4). The most significant reduction in WC and HC were observed in the AO group, while the most marked change in VFM was observed in C group.

**Table 4:** Comparison of anthropometric parameters in the RO, AO and C groups at the beginning of the study and the end of study (EOS)

Variable	RO group (n = 26)				AO group (n = 26)				C group (n = 29)				***P***
	Before	After	Δ	***P***	Before	After	Δ	***P***	Before	After	Δ	***P***	
Weight, kg	120.5 (102.9–135)	114.9 (97.7–128.2)	−5.6	**0.000**^**d**^	118.5 (108.0–132.4)	111.9 (106.5–126.0)	−6.6	**0.000**^**d**^	115.0 (101.4–133.3)	108.0 (96.4–127.0)	−7.0	**0.001**^**d**^	0.100^b^
BMI, kg/m^2^	39.8 (36.9–44.0)	39.1 (34.9–41.9)	−0.7	**0.001**^**d**^	40.6 (36.7–44.3)	38.9 (35.6–43.3)	−1.7	**0.000**^**d**^	38.4 (36.1–41.7)	37.7 (34.6–40.1)	−0.7	**0.000**^**d**^	0.204^b^
WC, cm	126.3 ± 13.3	123.2 ± 13.3	−0.4	**0.000**^**c**^	124.3 ± 17.2	120.5 ± 16.8	−3.8	**0.000**^**c**^	122.3 ± 20.6	121.1 ± 20.1	−1.2	**0.001**^**c**^	**0.022**^**a**^
HC, cm	128.0 ± 13.3	125.9 ± 13.3	−2.1	**0.000**^**c**^	130.6 ± 12.9	125.5 ± 11.6	−5.1	**0.000**^**c**^	119.9 ± 20.6	118.3 ± 19.5	−1.6	**0.000**^**c**^	**0.007**^**a**^
WHR	0.99 ± 0.09	0.99 ± 0.11	0.0	0.852^d^	0.95 ± 0.1	0.96 ± 0.11	+ 0.01	0.201^d^	1.02 ± 0.06	1.03 ± 0.05	−0.01	0.072^d^	0.064^b^
FM, %	41.0 ± 5.4	40.1 ± 5.7	−0.9	**0.031**^**c**^	42.1 ± 6.1	41.3 ± 7.1	−0.8	0.142^c^	40.9 ± 6.7	39.1 ± 6.9	−1.8	**0.000**^**c**^	0.112^a^
FM, kg	46.9 (41.9–56.2)	44.9 (36.8–52.6)	−2.0	**0.002**^**d**^	49.7 (41.0–59.6)	46.2 (37.1–56.9)	−3.5	**0.001**^**d**^	46.7 (40.4–54.1)	41.0 (37.2–51.4)	−5.7	**0.000**^**d**^	0.381^b^
LBM, kg	66.9 (58.6–75.6)	63.6 (55.2–74.8)	−3.3	**0.006**^**d**^	68.7 (56.5–77.2)	64.3 (56.9–76.5)	− 4.4	**0.002**^**d**^	70.1 (58.5–80.4)	64.5 (55.1–76.9)	−5.6	**0.000**^**d**^	0.894^b^
VFM, n	15.0 (12.0–21.0)	14.0 (11.0–20.0)	−1.0	**0.001**^**d**^	14.0 (13.0–21.0)	13.5 (12.0–21.0)	−0.5	**0.030**^**d**^	16.0 (12.0–20.0)	14.0 (11.0–18.0)	−2.0	**0.000**^**d**^	**0.020**^**b**^
TBW, %	41.6 (40.3–44.5)	43.2 (40.5–45.1)	+ 1.6	**0.010**^**d**^	41.4 (38.7–44.7)	42.8 (39.5–47.6)	+ 1.4	**0.014**^**d**^	41.8 (39.0–44.9)	42.0 (40.2–46.3)	+ 0.2	**0.000**^**d**^	0.374^b^
TBW, kg	49.1 (43.7–58.9)	47.9 (41.3–56.9)	−1.2	**0.014**^**d**^	50.6 (42.6–58.1)	48.5 (40.6–59.0)	−2.1	**0.012**^**d**^	48.6 (41.6–57.0)	49.1 (41.2–58.7)	+ 0.5	0.941^d^	0.302^b^
ECW, kg	22.7 ± 2.9	22.4 ± 3.2	−0.3	0.142^d^	23.1 ± 4.2	22.5 ± 4.1	− 0.6	**0.010**^**d**^	22.1 ± 3.5	21.9 ± 3.5	− 0.2	0.150^d^	0.261^b^
ICW, kg	25.6 (23.0–32.9)	25.1 (22.2–31.8)	− 0.5	0.121^d^	27.9 (22.5–33.5)	25.8 (21.9–31.6)	−2.1	**0.009**^**d**^	26.0 (21.4–31.8)	26.6 (21.6–32.0)	+ 0.6	0.241^d^	0.142^b^

Normally distributed data are represented by mean ± SD and skewed data by the median (interquartile range). *P* < 0.05 is considered significant (bold highlights)

^a^one way ANOVA, ^b^Kruskal-Wallis test, ^c^t- Student’s test, ^d^Wilcoxon test,

*Abbreviations*: *BMI* body mass index, *ECW* extracellular water, *EOS* end of study, *FM* fat mass, *HC* hip circumference, *ICW* intracellular water, *LBM* lean body mass, *TBW* total body water, *VFM* visceral fat mass, *WC* waist circumference, *WHR* waist-to-hip ratio

At the end of study, no significant improvements were observed in the clinical parameters of group C, whereas reductions in fasting insulin and HOMA-IR were observed in RO and AO groups. However, a significant reduction in fasting glucose levels was noted only in the AO group. Similarly, improvements in lipid parameters (TC, non-HDL, LDL, and TG) were also observed only in the AO group. An increase in HDL% was seen in both AO and RO groups. Furthermore, the results revealed significant differences in HOMA-IR between RO, AO and C groups (Table 5).

**Table 5:** Comparison of clinical parameters in the RO, AO, and C groups at the beginning of the study and the end of study (EOS)

Variable	RO group (***n*** = 26)				AO group (***n*** = 26)				C group (***n*** = 29)				***P***
	Before	After	Δ	***P***	Before	After	Δ	***P***	Before	After	Δ	***P***	
Fasting plasma glucose, mg/dL	105.0 (98.0–119.0)	103.5 (97.0–111.0)	−1.5	0.092^d^	110.5 (95.0–131.0)	102.0 (94.0–110.0)	−8.5	**0.034**^**d**^	104.0 (98.0–114.0)	104.5 (96.0–111.0)	+ 0.5	0.410^d^	0.130^b^
Fasting insulin, μU/L	18.5 (11.3–29.3)	12.6 (9.7–28.3)	−5.9	**0.001**^**d**^	22.6 (13.2–27.5)	16.9 (9.9–22.6)	− 5.7	**0.005**^**d**^	15.5 (13.0–22.2)	16.8 (12.4–26.1)	+ 1.3	0.481^d^	0.421 ^b^
HOMA-IR	4.8 (2.9–7.5)	3.7 (2.4–6.9)	−1.1	**0.002**^**d**^	6.1 (3.2–8.9)	5.6 (2.6–6.6)	−0.5	**0.031**^**d**^	4.0 (3.3–5.3)	4.4 (3.1–6.6)	+ 0.4	0.132^d^	**0.000**^**b**^
TC, mg/dL	196.6 ± 39.5	192.5 ± 32.4	−4.1	0.491^c^	193.8 ± 37.0	179.2 ± 32.7	−14.6	**0.032**^**c**^	192.5 ± 47.8	179.9 ± 38.1	−12.6	0.261^c^	0.641^a^
HDL, mg/dL	48.0 (41.0–66.0)	51.0 (43.0–61.0)	+ 3.0	0.372^d^	42.5 (37.0–48.0)	44.5 (38.0–51.0)	+ 2.0	0.152^d^	49.0 (42.0–57.0)	48.0 (44.0–54.0)	−1.0	0.142^d^	0.342^b^
HDL, %	29.2 (19.0–33.)	30.6 (21.0–33.2)	+ 1.4	**0.051**^**d**^	21.1 (18.0–29.6)	24.7 (20.8–31.4)	+ 3.6	**0.005**^**d**^	26.0 (22.0–32.0)	28.0 (24.0–36.0)	+ 2.0	0.443^d^	0.341^b^
non-HDL, mg/dL	143.5 ± 39.8	137.8 ± 33.6	−5.7	0.263^c^	149.6 ± 39.7	133.7 ± 34.1	−15.9	**0.010**^**c**^	149.1 ± 43.9	128.6 ± 36.9	−20.5	0.061^c^	0.371^a^
TG/HDL ratio	2.3 (1.4–4.0)	2.2 (1.5–3.2)	−0.1	0.550^d^	3.7 (2.4–5.4)	3.1 (1.9–4.7)	−0.6	**0.031**^**d**^	2.9 (2.0–5.6)	2.5 (1.6–3.1)	−0.4	0.173^d^	0.761^b^
LDL, mg/dL	121.5 ± 33.9	110.4 ± 35.1	−11.1	0.131^c^	117.6 ± 35.1	105.3 ± 30.2	−12.3	**0.042**^**c**^	108.0 ± 36.6	100.9 ± 33.5	−7.1	0.441^c^	0.821^a^
TG, mg/dL	97.0 (79.0–177.0)	120.0 (93.0–155.0)	−23.0	0.662^d^	143.0 (103.0–204.0)	136.5 (94.0–170.0)	−6.5	**0.000**^**d**^	142.0 (104.0–196.0)	133.0 (95.0–148.0)	−9.0	0.090^d^	0.600^b^

Normally distributed data are represented by mean ± SD and skewed data by the median (interquartile range). *P* < 0.05 is considered significant (highlighted by bold)

^a^one-way AOVA, ^b^Kruskal-Wallis test, ^c^t- Student’s test, ^d^Wilcoxon test,

*Abbreviations*: *EOS* end of study, *HDL* high-density lipoprotein cholesterol, *HOMA-IR* homeostatic model assessment of insulin resistance, *LDL* low-density lipoprotein cholesterol, *TC* total cholesterol, *TG* triglycerides

SI conversion factors: to convert TC, HDL, and LDL to mmol/L, multiply by 0.02586; TG to mmol/L, by 0.0114; glucose to mmol/L, by 0.05551

## Discussion

This is the first study which directly compared the effects of RSO or ASO supplementation on weight loss, as well as anthropometric and metabolic parameters, of obese participants of a weight loss program. RSO and ASO are characterized by a sizeable difference in fatty acid composition. ASO contains lower amounts of MUFAs (~ 24% vs. ~ 59%) and LC n-3 PUFAs (~ 1% vs. ~ 11%) and displays poorer PUFA/SFA and USFA/SFA ratios compared with those of RSO. Furthermore, ASO has a lower n-3/n-6 ratio than that of RSO [21]. By contrast, ASO is an oil with one of the highest squalene content. Strong anticancer, antioxidant, drug carrier, detoxifier, skin hydrating and emollient activities of squalene have been reported in both animal models and in vitro environments [22].

In contrast to studies based on long-term body mass reduction protocols, the current study evaluated whether a short-term weight loss program, conducted under strictly controlled conditions, would induce satisfactory anthropometric and metabolic changes in adult patients with obesity.

A previous study showed that the replacement of usual edible oils with oils rich in USFAs, such as ASO and RSO, resulted in lipid-modulating, anti-atherogenic, antioxidative, anti-inflammatory, hepatoprotective and hypotensive effects [7, 12]. The properties of dietary fat reportedly modulated obesity by interacting with genes encoding fatty acid metabolism, adipogenesis and endocannabinoid system [23].

Animal studies have indicated that LC n-3 PUFAs may protect against weight gain, raising the possibility that LC n-3 PUFA facilitates weight loss or differential changes in body composition when incorporated into weight-loss programs [23]. Furthermore, Borsonelo et al. [24] demonstrated anxiolytic-like effects of PUFA enriched diets on animal anxiety models. A time-dependent effect of LC n-3 PUFAs on weight loss in humans has been reported [25, 26]. Certain studies have shown that MUFAs that induce higher energy expenditure, diet-induced thermogenesis, and fat oxidation than that by PUFA diets, may affect weight loss more effectively than PUFAs [27, 28]. However, the current study was unable to confirm whether oil supplementation during a weight reduction program increases the effectiveness of interventions. At the end of the study, significant reductions in weight, BMI, WC, HC, and FM were observed in each group, and also in subjects from the control group. Except for WC, HC and VFM, no significant differences were observed in weight loss or other anthropometric parameters between groups. Weight loss and improvement in body composition observed in this study were comparable with those observed in other studies [29, 30]. It is noteworthy that the most significant reduction in VFM was observed in patients without oil supplementation, although it did not result in an improvement in metabolic parameters.

Numerous studies have indicated that consumption of high levels of MUFAs and PUFAs may improve glucose metabolism and lipid profile, compared to the consumption of fats containing higher levels of SFAs. However, whether replacement of dietary SFAs with higher concentrations of MUFAs or PUFAs would further enhance metabolic parameters remains unclear [31, 32]. A meta-analysis conducted by Qian et al. [33] revealed that, consumption of MUFA-rich diets resulted in significant reduction in fasting plasma glucose and a nonsignificant reduction in fasting insulin, TG, and LDL levels, compared to consumption of high-PUFA diets. By contrast, Miller et al. [32] demonstrated that substituting SFA with PUFAs in metabolic syndrome patients resulted in a higher reductions of TG and improved endothelial function than MUFAs.

The current study did not indicate any differences between intervention induced changes in the clinical parameters of AO, RO and C, except in HOMA-IR. HOMA-IR was most markedly reduced in the OR group, while an increase in HOMA-IR was noticed in the C group. However, there was a trend toward significantly reduced fasting serum insulin levels and HDL% in AO and RO groups, as opposed to the C group. Additionally, statistically significant changes in the fasting glucose level, TC, non-HDL, TG/HDL ratio, LDL and TG were observed in the AO group.

Previous studies have suggested that RSO may be used to normalize glucose profiles in humans [34–36]. A study of type 2 diabetes patients treated with an oral antihyperglycemic agent at a Canadian academic center, showed that consuming a canola oil-enriched low-glucose diet for 3 months improved glycemic control [34]. The effect of ASO on glucose metabolism was less clear. Kim et al., showed that 3 weeks of ASO supplementation (100 mg/kg) significantly reduced serum glucose levels in streptozocin-induced diabetic rats [11]. The beneficial effect of ASO in patients with diabetes mellitus type 2 has also been confirmed by Miroshnichenko et al. [37]. The current study observed significant improvements in fasting insulin levels and insulin sensitivity in the AO and RO groups, although changes in glucose levels were observed only in subjects supplemented with ASO.

The effect of RSO on circulatory cholesterol levels has been reported in most short-term interventions [38]. Lin et al. [7] demonstrated that diets rich in RSO resulted in substantial reductions in TC (12.2–12.5%) and LDL levels (17%). However, changes induced in HDL and TG levels by canola oil were evidently inconsistent. Furthermore, previous studies have reported that compared to consumption of high-SFA diets, consumption of diet enriched with RSO resulted in 8–10% reduction in HDL concentrations [39–41]. Data from this study showed that a calorie-restricted RSO-supplemented diet does not significantly affect TC and TG levels. A slight increase in HDL concentration and improvement in non-HDL and TG/HDL ratios were observed, but these changes were not statistically significant.

The beneficial effect exerted by ASO on cholesterol and bile acid absorption, cholesterol lipoprotein distribution, hepatic cholesterol content and cholesterol biosynthesis was demonstrated by Berger et al. [42], via an animal model study. In this study, hamsters were given hypercholesterolemic diets consisting of control, 10, or 20% *Amaranthus cruentus* grain, or 2.5 or 5% crude amaranth oil for 4 weeks. The results showed that amaranth oil (5%) significantly decreased TC HLD and VLDL, compared to the control, and increased fecal excretion of particular neutral sterols and the bile acid, ursodeoxycholate [42]. However, an animal study [43] and a human pilot study [44] conducted by Berbger et al., revealed that cholesterol-lowering properties of ASO did not affect lipid profiles in an identical manner, and that the final effect of ASO on cholesterol metabolism may depend on factors such as amaranth species and cultivars, growing and processing conditions, as well as unique nutritional compositions.

Gonor et al. [45], investigated the beneficial effects exerted by a diet supplemented with squalene (600 mL/d) from amaranth oil (18 mL/d) on TC and TG concentrations and the fatty acid composition of erythrocytes, in patients with ischemic heart disease and hyperlipoproteinemia. Similarly, Martirosyan et al. [46], showed that 3 weeks of exposure to low-sodium/low-fat diets containing ASO (3, 6, 12, or 18 mL/d) promoted positive dose-dependent changes in serum TC, LDL and TG levels in obese patients with coronary heart disease and hypertension.

The current study revealed that a 3-week intervention with ASO supplementation (20 mL/d) led to a significant reduction in TC, %HDL, LDL, and TG levels and caused a slight, nonsignificant, increase in HDL levels. Statistically significant improvements in non-HDL and TG/HDL levels were also observed in the AO group. Although, ASO contains lower amounts of MUFA and LC n-3 PUFA than RSO, the presented study demonstrated that ASO caused more marked changes in lipid profiles than RSO. A better understanding of reasons underlying this finding may require further investigation.

The results of the current study showed that supplementation with ASO and RSO during the 3-week body mass reduction program did not cause changes in anthropometric measurements and clinical outcomes that were more effective, compared to group C. However, the study revealed a trend toward a more marked improvement in carbohydrate and lipid profiles in AO and RO groups compared with that in group C.

## Study strengths and limitations

The strength of this study is that the intervention was conducted under strictly controlled conditions. During the 3-weeks program, the participants were in the hospital ward, being under constant control over diet, supplementation and physical activity. The major limitations of this trial were small sample size and the relatively short duration of intervention. The main reason for such a short duration was that continued hospitalization for 3 weeks may interrupt professional and personal activities. However, it is noteworthy that presented study was one of the few studies conducted under specific and strictly controlled conditions, which is rare in nutritional interventions. Participants of the study received the same type of hypocaloric diet prepared by a dietetic food caterer and underwent the same physical activity program with a physical therapist. The 3-week hospitalization allowed the involvement of the study population in the intervention to be controlled. Although dual energy X-ray absorptiometry (DXA) is the gold standard for the assessment of body composition, the bioimpedance method (BIA) was used in the study due to its non-invasiveness, lower cost, and widespread use.

## Conclusions

The results of this study indicated that although the 3-week diet and physical activity program exerted a significant effect on anthropometric and clinical parameters, this effect was independent of ASO or RSO supplementation. However, ASO or RSO supplementation resulted in a trend toward more marked changes in the carbohydrate and lipid profiles of AO and RO groups, compared with those of the C group. The use of therapies involving edible oils with high nutritional value, such as RSO and ASO, during body mass reduction programs, may enhance metabolic measurements.

## Data Availability

The datasets used and analyzed during the current study are available from the corresponding author on reasonable request.

## Abbreviations

AO: Amaranth seed oil group
ASO: Amaranth seed oil
BIA: Bioimpedance
BMI: Body mass index
C: Control group
DXA: Dual X-ray absorptiometry
ECW: Extracellular water
FM: Fat mass
HC: Hip circumference
HDL: High-density lipoprotein
HOMA-IR: Homeostatic model assessment of IR
ICW: Intracellular water
LBM: Lean body mass
LC n-3 PUFAs: Long chain polyunsaturated fatty acids
LDL: Low-density lipoprotein
MUFA: Monounsaturated fatty acids
RO: Rapeseed oil group
PAL: Physical activity level index
PUFA: Polyunsaturated fatty acids
RSO: Rapeseed oil
SFA: Saturated fatty acids
TBW: Total body water
TC: Total cholesterol
TDEE: Total daily energy expenditure
TG: Triglycerides
USFA: Unsaturated fatty acids
VFM: Visceral fat mass
WC: Waist circumference
WHO: World Health Organization
WHR: Waist-to-hip ratio
